# Characteristics and prognosis of language impairment in subcortical aphasia of acute stroke patients

**DOI:** 10.3389/fneur.2025.1630365

**Published:** 2025-07-16

**Authors:** Zinan Yuan, Siqi Li, Xinya Chen, Yang Liu, Anji Zheng, Liqun Gao, Zaizhu Han, Yumei Zhang

**Affiliations:** ^1^Department of Neurology, Beijing Tiantan Hospital, Capital Medical University, Beijing, China; ^2^The Rehabilitation Department, Beijing Xiaotangshan Hospital, Beijing, China; ^3^Beijing Language and Culture University, Beijing, China; ^4^State Key Laboratory of Cognitive Neuroscience, Beijing Normal University, Beijing, China; ^5^Department of Rehabilitation Medicine, Beijing Tiantan Hospital, Capital Medical University, Beijing, China

**Keywords:** subcortical aphasia, lexical-semantic processing, phonological processing, language recovery, Chinese Aphasia Language Battery, principal component analysis

## Abstract

**Background:**

Subcortical aphasia, caused by lesions in deep brain structures such as the basal ganglia, thalamus, and periventricular white matter, remains poorly understood due to its heterogeneous clinical presentations and disputed neural mechanisms. Unlike classical cortical aphasia syndromes, subcortical aphasia often involves subtle deficits in lexical, semantic, and phonological processing, which may be underestimated by standard assessments.

**Objective:**

This study aimed to comprehensively characterize the language profiles of patients with subcortical aphasia using a multidimensional assessment approach, and to explore the underlying components of language impairment and their relationship to aphasia severity.

**Methods:**

Thirty-four right-handed, native Chinese-speaking patients with first-ever, MRI-confirmed subcortical stroke and aphasia were enrolled within 4 weeks post-stroke. Standardized assessments included the Chinese version of the Western Aphasia Battery (WAB), the Aphasia Severity Rating Scale (ASRS), the Chinese Aphasia Fluency Characteristic Scale, and the naming battery of Chinese Aphasia Language Battery (CALB-nb). Principal component analysis (PCA) and correlation analyses were used to identify key dimensions of language impairment, with correlation coefficients calculated to quantify patient performance across linguistic domains. A one-year follow-up assessment was conducted using the ASRS to evaluate prognostic outcomes of the enrolled patients.

**Results:**

Most patients exhibited mild to moderate aphasia, with anomic aphasia being the most prevalent subtype (47.1%). CALB naming battery results revealed high accuracy in tone decoding but lower performance in low-frequency word performance and semantic association. Strong correlations were found between phonological output and both auditory perception and phonemic decoding, as well as between auditory lexical comprehension and multiple semantic tasks. PCA identified two components—lexical-semantic and phonological-auditory, which together explained 77.3% of the variance. A composite PCA score significantly predicted aphasia severity (*R*^2^ = 0.31, *p* < 0.001). At one-year follow-up, 73.6% of patients achieved functional language recovery (ASRS 4–5), and five patients resumed their pre-stroke occupations.

**Conclusion:**

Multidimensional assessments reveal distinct but interrelated components of lexical-semantic and phonological processing, which are closely linked to functional recovery. These findings underscore the necessity for sensitive and domain-specific language evaluations to inform prognosis and guide individualized rehabilitation strategies for subcortical aphasia.

## Introduction

Aphasia is a clinical syndrome characterized by acquired impairments in language production and comprehension, most commonly caused by damage to the cortico-subcortical language networks in the left hemisphere (1). Epidemiological data indicate that approximately 21–40% of stroke survivors experience persistent aphasia (2), which significantly impairs their functional independence and quality of life (3). While cortical damage (particularly to perisylvian language areas) has long been recognized as the primary source of aphasic symptoms, increasing evidence highlights that subcortical lesions can also result in clinically significant language deficits (4, 5). However, subcortical aphasia remains underexplored, owing to its heterogeneous symptom profiles, subtle presentations, and the lack of well-defined mechanistic frameworks (6).

Subcortical structures such as the basal ganglia, periventricular white matter, and the thalamus are thought to contribute to language via their extensive connections with cortical language areas (7). The basal ganglia, a highly interconnected neural network implicated in both motor regulation and higher-order cognitive behaviors, have been proposed to play a role in speech initiation through their connections with the pre-supplementary motor area (pre-SMA) (8). Lesions in this region often produce dysfluent speech, prosodic disturbances, or anomic symptoms (9, 10). Damage to the periventricular white matter may disrupt language processing by interrupting the connectivity between Broca’s and Wernicke’s areas, leading to disconnection syndromes that resemble anomic or transcortical aphasia (11). The thalamus plays a unique role in lexical selection, semantic regulation, and attentional control (4). Thalamic aphasia, despite its rarity, presents with fluent, paraphasic speech, frequent perseverations, and relatively preserved repetition (12).

Although prior studies have attempted to classify subcortical aphasia into thalamic and non-thalamic subtypes, such lesion-localization approaches may oversimplify the complex cortico-subcortical integration underlying language (13–16). As language functions are governed by a complex, interrelated network encompassing the cortex, basal ganglia, thalamus, and cerebellum, any focal lesion within this circuit may disrupt overall language processing (17). Behavioral overlap across different lesion sites suggests that phenotypic convergence is common (11, 16), with many patients demonstrating mild symptoms and favorable recovery trajectories (6). This underscores the need to add functionally grounded assessments that better capture the multidimensional nature of language deficits.

While neuroimaging modalities such as functional magnetic resonance imaging (fMRI) or diffusion tensor image (DTI) provide spatial resolution and connectivity data (18, 19), these studies still lack comprehensive behavior assessment and may not reliably reflect functional communication abilities in real-world clinical settings. In contrast, standardized language assessments remain the most accessible and direct tools for characterizing linguistic deficits, particularly in acute-care or resource-limited environments. These tools can detect impairments that are behaviorally relevant but not always visible on imaging, especially in cases of thalamic aphasia where higher-order functions such as semantic control or verbal fluency are selectively affected.

Subcortical aphasia is frequently characterized by semiological features that do not conform to the classical aphasia syndromes outlined in the Wernicke-Geschwind model (20). For instance, the thalamus is specifically involved in higher-order language functions, and impairments resulting from thalamic lesions are often subtle, thereby escaping detection by standard language assessments (1). However, current aphasia batteries are often based on Indo-European languages and may lack sensitivity to the unique linguistic and cognitive features of Mandarin Chinese, including tonal processing, Chinese verb structure, and homophones discrimination. Furthermore, many tools do not provide sufficient granularity to detect subtle dissociations across phonological, lexical, and semantic domains (21). This limitation may contribute to underdiagnosis or mischaracterization of subcortical aphasia in Mandarin-speaking populations. As a result, subcortical aphasia may remain underdiagnosed even when conventional diagnostic tools are applied. This underscores the need for more sensitive assessment instruments or domain-specific evaluations that target discrete components of language function, particularly when evaluating thalamic involvement (1).

To address this gap, we employed the Chinese Aphasia Language Battery (CALB), a comprehensive and theory-driven assessment co-developed by Beijing Language and Culture University and Northwestern University. Grounded in psycholinguistic models of lexical access and sentence processing (22), the CALB is the first Mandarin-based tool specifically designed to assess phonological, lexical, syntactic, and semantic functions within a Chinese linguistic context. It is suitable for both stroke-related and neurodegenerative aphasia and is particularly effective in identifying fine-grained impairments across multiple language domains.

This study represents the first clinical application of the CALB in a cohort of Mandarin-speaking patients with subcortical stroke, offering a novel perspective on the cognitive-linguistic mechanisms underlying subcortical aphasia. By combining componential behavioral profiling with exploratory multivariate analysis, our aims were to: (1) delineate distinct patterns of language performance across phonological, lexical, and semantic domains; (2) identify latent factors underlying performance variability; and (3) explore the prognosis of patients with subcortical aphasia.

## Methods and material

### Participants

A total of 34 patients with subcortical aphasia (24 males, 73.5%) were enrolled, with a mean age of 52.4 years old and an average of 16 days since stroke onset at the time of evaluation. Of these, 9 were basal ganglia lesions, 3 thalamus, 17 deep white matter lesions and 5 mixed lesions (Table 1). All enrolled patients completed the baseline evaluation. The patients were recruited at Neurology Center and Department of Rehabilitation Medicine, Beijing Tiantan Hospital, Capital Medical University.

**Table 1:** Characteristic of the enrolled patients.

Characteristic	Value
Age (mean, SD)	52.44, 10.45
Males (%)	25 (73.53%)
Time post onset (mean, SD)	16.44 (10.42)
Stroke type, *n* (%)	
Ischemic stroke	22 (64.71%)
Hemorrhagic stroke	12 (35.29%)
Stroke location	
Basal ganglia	9 (26.47%)
Thalamus	3 (8.82%)
Deep white matter	17 (50.00%)
Mixed subcortical areas	5 (14.71%)
Fluent type, *n* (%)	
Fluent	19 (55.88%)
Mild	3 (8.82%)
Non-fluent	12 (35.30%)
Aphasia Quotient (mean, SD)	58.24, 22.37
Aphasia type, *n* (%)	
Broca’s aphasia	10 (29.41%)
Wernicke’s aphasia	4 (11.76%)
Anomic aphasia	16 (47.06%)
Conduction aphasia	1 (2.94%)
Transcortical sensory aphasia	1 (2.94%)
Global aphasia	2 (5.88%)
ASRS score (baseline), *n* (%)	
0	1 (2.94%)
1	10 (29.41%)
2	10 (29.41%)
3	13 (38.24%)
ASRS score (1 year), *n* (%)	
1	1 (2.94%)
2	1 (2.94%)
3	4 (11.76%)
4	11 (32.35%)
5	14 (41.18%)

The inclusion criteria were as follows: ① Native Chinese speakers with at least 6 years of formal education and right-handedness. ② Age between 18 and 80 years. ③ First-ever stroke confirmed by structural magnetic resonance imaging (MRI), with lesions confined exclusively to subcortical regions. ④ Aphasia secondary to acute stroke (onset <30 days), defined by Aphasia Quotient (AQ) below 93.8 on the Chinese version of the Western Aphasia Battery (WAB) and an Aphasia Severity Rating Scale (ASRS) score below 3 on the Boston Diagnostic Aphasia Examination (BDAE). ⑤ Clinically stable neurological status at the time of enrollment. Exclusion criteria included a history of pre-existing language disorders, cognitive impairment, a diagnosis of untreated mental illness prior to stroke onset (based on self-report), significant visual or auditory impairments, or the presence of extensive white matter hyperintensities of presumed vascular origin (Fazekas score of 2–3 on MRI). The study protocol was approved by the Ethics Committee of Beijing Tiantan Hospital, Capital Medical University (Approval Number: KY2024-156-02), and written informed consent was obtained from all participants prior to their inclusion.

### Behavioral evaluation indicators of language function

#### General language assessment

All enrolled patients received general language assessment within 3 days. Initially, the Chinese version of the WAB was administered to calculate the AQ and classify aphasia types (23). This battery is a commonly used general assessment tool in clinical practice for aphasia. It enables preliminary quantification of the aphasia severity and facilitates classification of the aphasia type. The current study adopted four oral language subtests from the WAB: spontaneous speech (including information content and fluency), auditory comprehension, repetition, and naming. The AQ, derived from the patients’ performance in these subtests, served as an indicator of aphasia severity. To further evaluate speech fluency, the Chinese Aphasia Fluency Characteristic Scale was used. Based on articulatory agility, intonation/melodic line, grammatical well-formedness, and phrase length, speech output was categorized as fluent, mildly fluent, or non-fluent type. Functional language ability was assessed using the ASRS of the BDAE, which ranges from scale 0 (no usable speech or auditory comprehension) to scale 5 (minimal discernible speech handicap), with higher scores indicating better functional communication. The ASRS is a widely used clinical scale for grading aphasia severity. It demonstrates good reliability and validity, primarily assessing patients’ spoken language production capacity (24). This tool provides a simple evaluation while effectively reflecting their functional language performance in real-world contexts.

#### Comprehensive neuropsychological test

Subsequently, patients underwent an extended language assessment using the CALB naming battery (CALB-nb), which comprises eight subtasks. Assessments were administered on a Lenovo YOGA TABLET (Lenovo YT3-X50F, Android 6.0.1), with all instructions automatically delivered by the device and supervised by a trained therapist. Before each subtask, 2–3 practice trials were conducted to ensure the patient fully understood the task requirements. Stimuli were presented visually or auditorily, and each subtask consisted of 12–70 test trials involving both target and distractor options. A correct response within the time limit was scored as correct; incorrect responses, mispronunciations, or timeouts were not scored. For patients with motor impairments preventing touchscreen use, the therapist assisted in response selection based on the patient’s indications. The eight subtasks included: auditory discrimination, tone recognition, auditory lexical decision, confrontation naming, auditory comprehension, semantic association, non-word repetition and word repetition. These tasks were designed to evaluate various stages of language processing, including phonological input, tonal and phonological decoding, lexical comprehension, semantic association, and both phonological and lexical output. Specific lexical processing abilities such as lexical categorization and verb argument structure were inferred based on performance across different lexical categories. As all tasks were presented using visual or auditory modalities, the patient’s visual and auditory perception abilities were also indirectly assessed based on their accuracy and response patterns. All assessments were conducted in a quiet speech and language therapy room (see Supplementary material for detailed assessment procedures).

### Follow-up procedure

A telephone follow-up was conducted 1 year after enrollment to assess patients’ functional speech using the ASRS. Each evaluation was performed by one therapist during a 10–20-min conversation with the patient, while two additional therapists observed simultaneously. Patients were encouraged to engage in spontaneous speech by responding to open-ended questions on topics such as recent daily activities, emotional states, interactions with caregivers, and the role of family in their language rehabilitation. All three therapists independently rated the patient’s functional speech using the ASRS, and the most frequently assigned score among the three was recorded as the final score.

### Data collation and statistical analysis

All statistical analyses were conducted using SPSS 26.0 and figures were created using Origin 2021 and GraphPad Prism 9.5. Continuous variables, including the WAB subtest scores and AQ, were analyzed as scaled variables. To facilitate comparison across subtests, individual WAB subtest scores were normalized to a 10-point scale. The AQ was calculated using the standard formula:

$\mathrm{AQ}=(\begin{array}{l} \text{Spontaneous Speech}+\text{Auditory Comprehension}\\ +\text{Repetition}+\text{Naming} \end{array})\times 2$

The AQ ranges from 0 to 100, with severity of aphasia classified according to established thresholds: mild (AQ 50.4–93.7), moderate (AQ 30.1–50.3), and severe (AQ 0–30.0), based on Chinese reference criteria (25). For the CALB-nb, raw scores from individual subtasks were converted into 11 distinct language domains: Phonemic Decoding (PD), Tone Decoding (TD), Auditory Lexical Comprehension (ALC), Phonological Output (PO), Phonological Lexical Production (PLP), Low-frequency Word performance (LfW), Noun Categorization (NC), Animacy Effects (AE), Noun-to-Verb Ratio (NVR), Verb Argument Structure (VAS), and Semantic Association (SA). In addition, task responses involving different sensory modalities were further classified into two perceptual domains: vision perception and auditory perception. Except for the NVR, all domain scores were expressed as accuracy rates ranging from 0 to 100, calculated as the percentage of correct responses out of total trials for each domain. NVR, by contrast, was defined as the ratio of correct responses in *noun-related tasks* to those in *verb-related tasks*, reflecting the relative strength of noun versus verb processing. Because NVR represents a proportional rather than bounded accuracy score, it was treated as a distinct metric in subsequent analysis and not included in the dimensionality reduction procedures. The ASRS scores and fluency types were treated as categorical variables.

Descriptive statistics were first computed to characterize the overall linguistic profile of participants. For continuous variables, means, standard deviations (SD), and ranges were reported. Frequencies and percentages were used to summarize categorical variables. Between-group comparisons were performed using independent sample t-tests for normally distributed continuous variables and Mann–Whitney U tests for non-parametric distributions. Categorical variables were compared using the chi-square test or Fisher’s exact test, as appropriate. For comparisons involving more than two groups, a mixed analysis of variance (ANOVA) was used, followed by Least Significant Difference (LSD) *post hoc* tests to identify pairwise differences.

To explore relationships among specific language domains, pairwise Pearson correlation analyses were conducted among the 11 CALB-nb domain accuracy. Correlation coefficients (*r*) and corresponding *p*-values were reported to assess the strength and significance of associations between domains. A *post hoc* power analysis was conducted using G*Power 3.1. To further explore the underlying structure of the language impairments observed and reduce dimensionality among the interrelated language domains, a principal component analysis (PCA) with varimax rotation was conducted across the 10 CALB-nb domains (except NVR). Eigenvalues, scree plots, and parallel analysis were used to determine the number of components to retain. Components with eigenvalues greater than 1.0 (Kaiser’s criterion) were initially retained, and the inflection point of the scree plot was used to confirm the number of meaningful components. Varimax rotation was applied to facilitate interpretability of factor loadings by maximizing the variance of squared loadings within each component. Following extraction and interpretation of the principal components, individual-level component scores were computed using the regression method, yielding standardized scores for each retained component. A composite language performance score (*x score*) was subsequently derived by calculating a weighted average of the retained principal component scores, reflecting a subject’s overall language functioning profile in a single, continuous variable. The weights were based on the proportion of variance explained by each component. Finally, a linear regression analysis was conducted to examine the relationship between the composite *x score* and AQ. In this model, AQ served as the dependent variable, and the composite *x score* was entered as the primary independent predictor. Additional variables, including age, sex, time post-onset, and aphasia subtype, were entered as covariates to control for potential confounding effects. All statistical tests were two-tailed, and a *p*-value < 0.05 was considered statistically significant unless otherwise specified.

## Results

### Language assessment result

**Figure 1:**
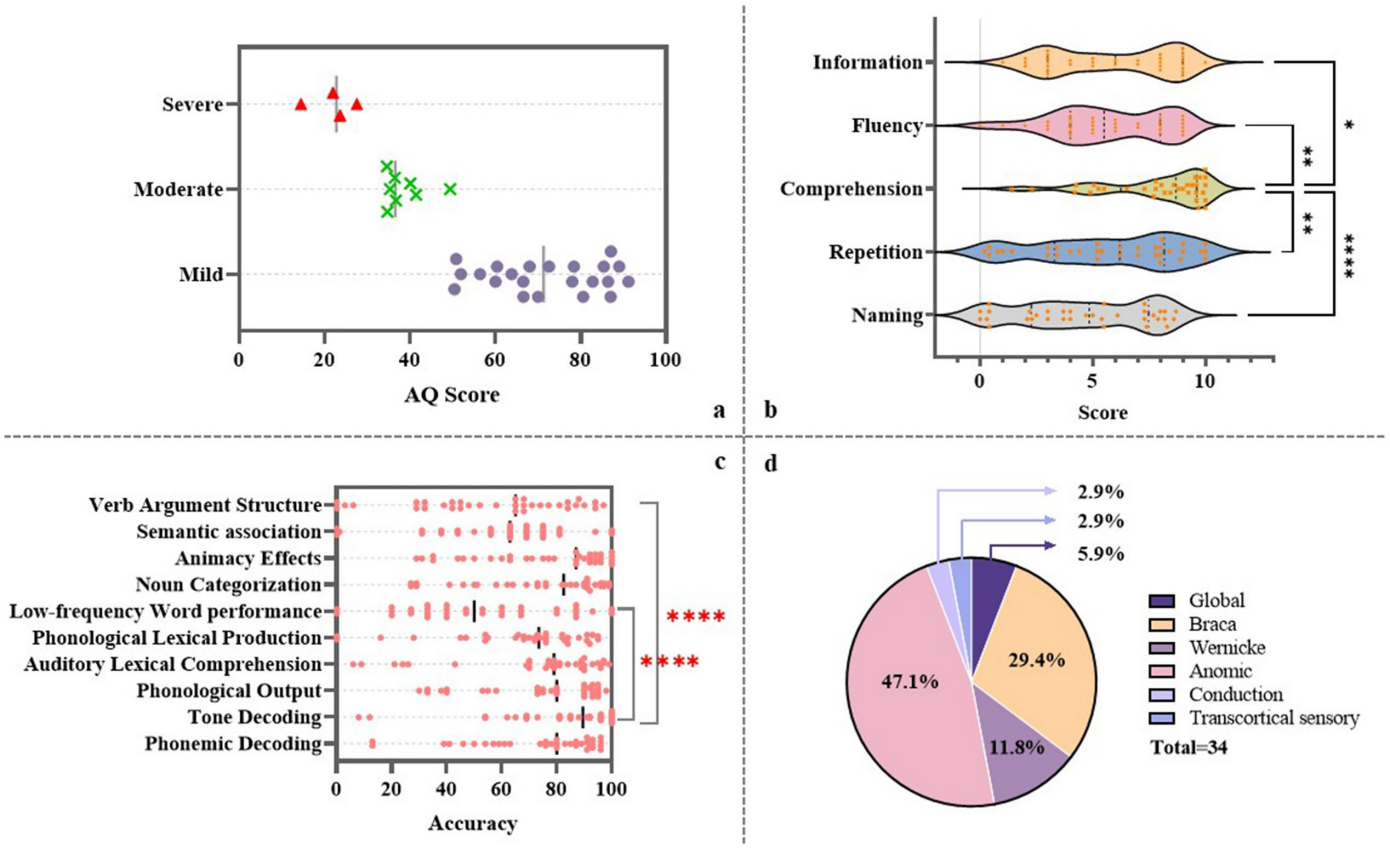
Language performance of patients with subcortical aphasia. **(a)** AQ scores across the cohort. **(b)** Group-level performance on subtests of the WAB. **(c)** Accuracy rates across the subtasks of the CALB-nb. **(d)** Distribution of aphasia types among enrolled patients.

Among the 34 enrolled patients, more than half were classified as having fluent aphasia (55.88%). In terms of aphasia type, nearly half were diagnosed with anomic aphasia (47.1%), followed by Broca’s aphasia, which accounted for approximately one-third of the cohort (29.4%) (Figure 1d). Regarding aphasia severity, the majority of patients presented with mild to moderate aphasia, with only four individuals classified as having severe aphasia (Figure 1a). In the WAB subtests, patients generally showed higher scores in auditory comprehension (*M* = 8.70, SD = 2.27, *p* < 0.05), whereas greater variability was observed in the repetition (*M* = 5.75, SD = 3.15) and naming (*M* = 4.68, SD = 2.92) subtests (Figure 1b).

**Figure 2:**
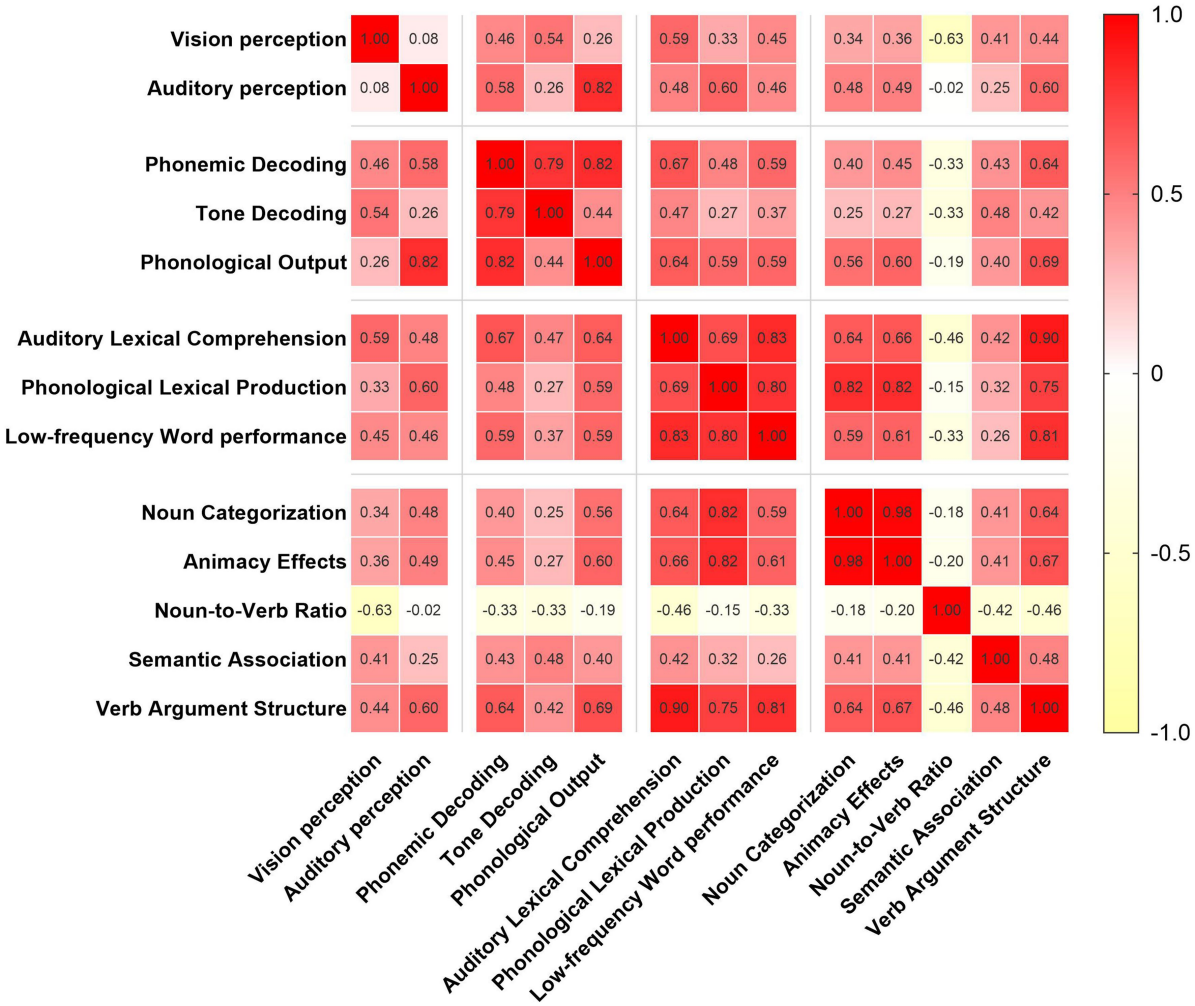
Correlation matrix of CALB-nb subtask accuracy.

In terms of performance across specific language processing domains, patients achieved the highest accuracy in the TD task (*M* = 81.65, SD = 23.00), while the lowest performance was observed in the LfW task (*M* = 52.59, SD = 27.75), followed by the VAS task (*M* = 56.53, SD = 28.47) (Figure 1c). Pearson correlation analyses revealed significant positive linear relationships between several language processing domains. Specifically, PO was strongly correlated with auditory perception (*r* = 0.819, *p* < 0.001, 95% CI: 0.66–0.91) and PD (*r* = 0.822, *p* < 0.001, 95% CI: 0.67–0.91). ALC was significantly associated with both VAS (*r* = 0.900, *p* < 0.001, 95% CI: 0.80–0.95) and LfW (*r* = 0.835, *p* < 0.001, 95% CI: 0.67–0.90). PLP showed strong correlations with LfW (*r* = 0.797, *p* < 0.001, 95% CI: 0.62–0.90), NC (*r* = 0.818, *p* < 0.001, 95% CI: 0.66–0.91), and AE (*r* = 0.821, *p* < 0.001, 95% CI: 0.66–0.91). In addition, LfW and VAS were also highly correlated (*r* = 0.810, *p* < 0.001, 95% CI: 0.65–0.90), as were NC and AE (*r* = 0.980, *p* < 0.001, 95% CI: 0.96–0.99) (Figure 2). *Post hoc* power analysis indicated 76% power to detect medium effects (*ρ* = 0.4) at *α* = 0.05 with *N* = 34, suggesting adequate sensitivity for primary hypothesis. These findings indicate a strong interdependence between phonological output, auditory perception, and phonemic decoding. Moreover, auditory lexical comprehension was closely associated with multiple lexical-semantic tasks, underscoring its central role in lexical-semantic processing. The positive correlations observed among various lexical and semantic tasks further suggest their intrinsic interconnectedness. Overall, the results illustrated in Figure 2 support the notion that language processing involves a dynamic, interrelated system in which different components mutually influence each other.

### PCA analysis of language processing domains

**Figure 3:**
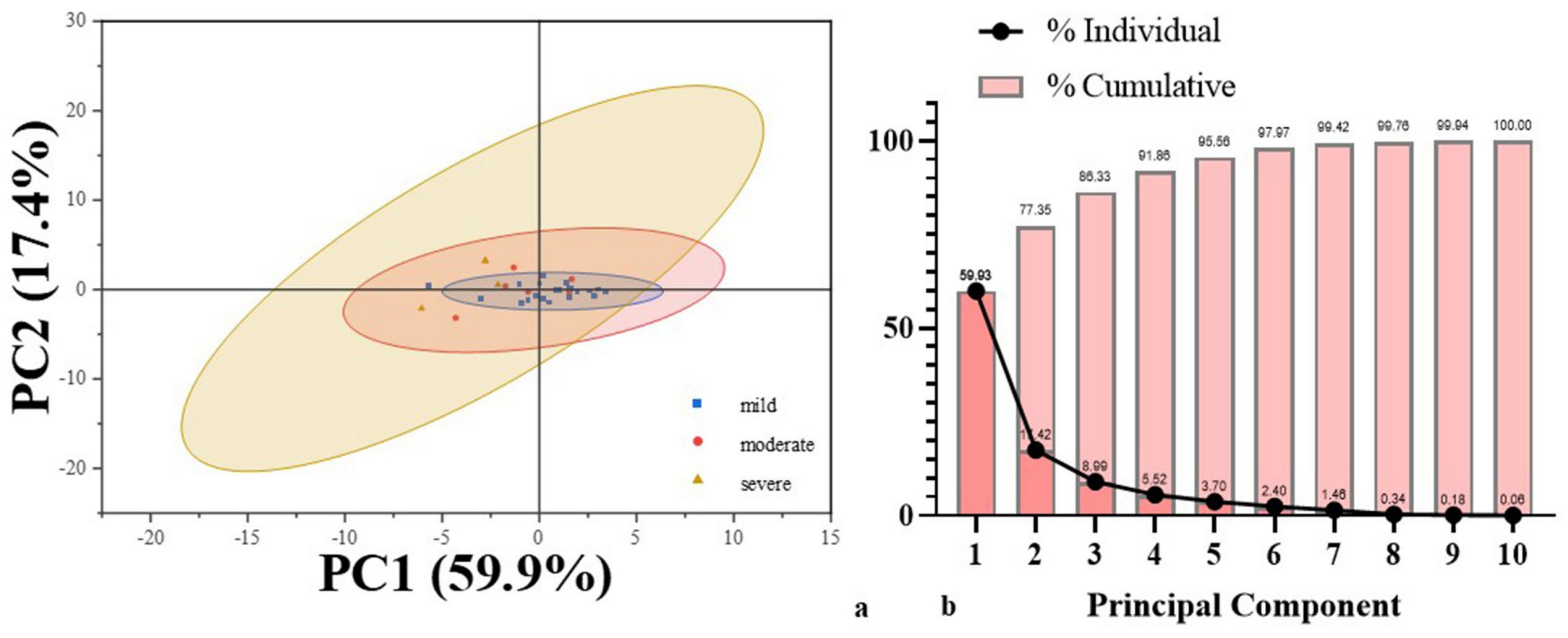
Results of PCA analysis. **(a)** PCA score plot illustrating individual patients projected onto the first two principal components. **(b)** Screen plot showing the proportion of variance explained by each principal component.

The Pearson correlation analysis revealed significant correlations among language domains. Subsequently, PCA was employed for dimensionality reduction of variables. Variables included in the analysis were derived from CALB-nb language task accuracy. Bartlett’s test of sphericity was significant (*p* < 0.001), and the Kaiser–Meyer–Olkin (KMO) measure of sampling adequacy was 0.66, indicating the data were appropriate for PCA. Two principal components with eigenvalues greater than 1 were extracted, cumulatively explaining 77.34% of the total variance in language performance (Figure 3). The first principal component (PCA1) accounted for 59.9% of the variance and was heavily loaded on PLP (0.92), NC (0.92) and AE (0.91). These variables primarily index lexical retrieval and semantic integration, suggesting that PCA1 represents a *lexical-semantic dimension* of language processing. The second principal component (PCA2), explaining 17.4% of the variance, and showed high loadings on PO (0.94), TD (0.95), ALC (0.80). This pattern reflects core aspects of phonological and auditory decoding, suggesting PCA2 captures a *phonological-auditory processing* dimension.

**Figure 4:**
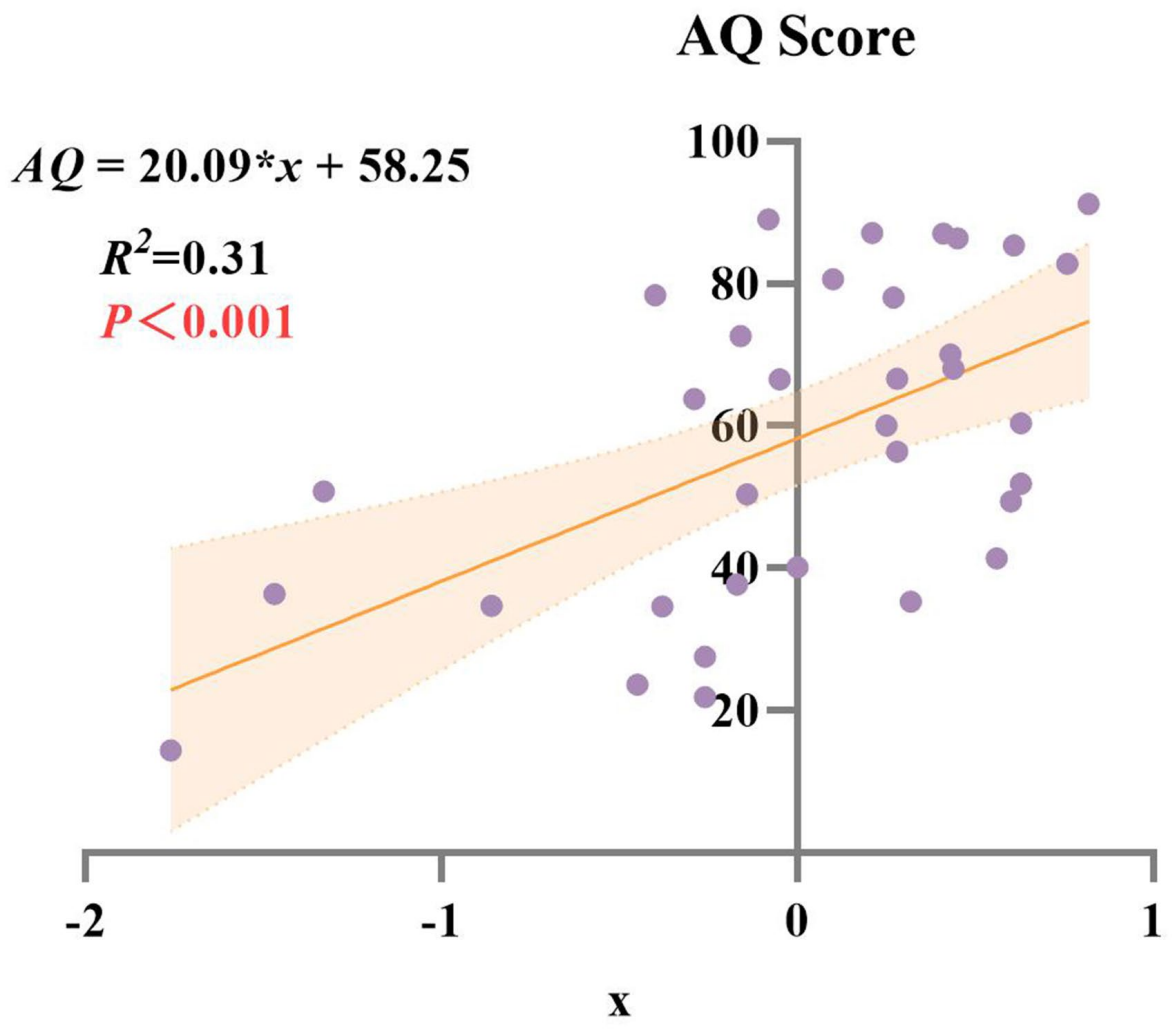
Linear regression analysis of AQ score and composite score (*x*).

A composite score (*x*) was calculated from the two principal components and used to examine its relationship with the AQ score. Linear regression analysis demonstrated a significant positive relationship between *x* and AQ (*R*^2^ = 0.31, *p* < 0.001), indicating that the composite score explained 31% of the variance in aphasia severity (Figure 4). This result highlights the utility of the principal components in capturing the overall severity of language impairment in this population.

### Prognosis of subcortical aphasia patients

**Figure 5:**
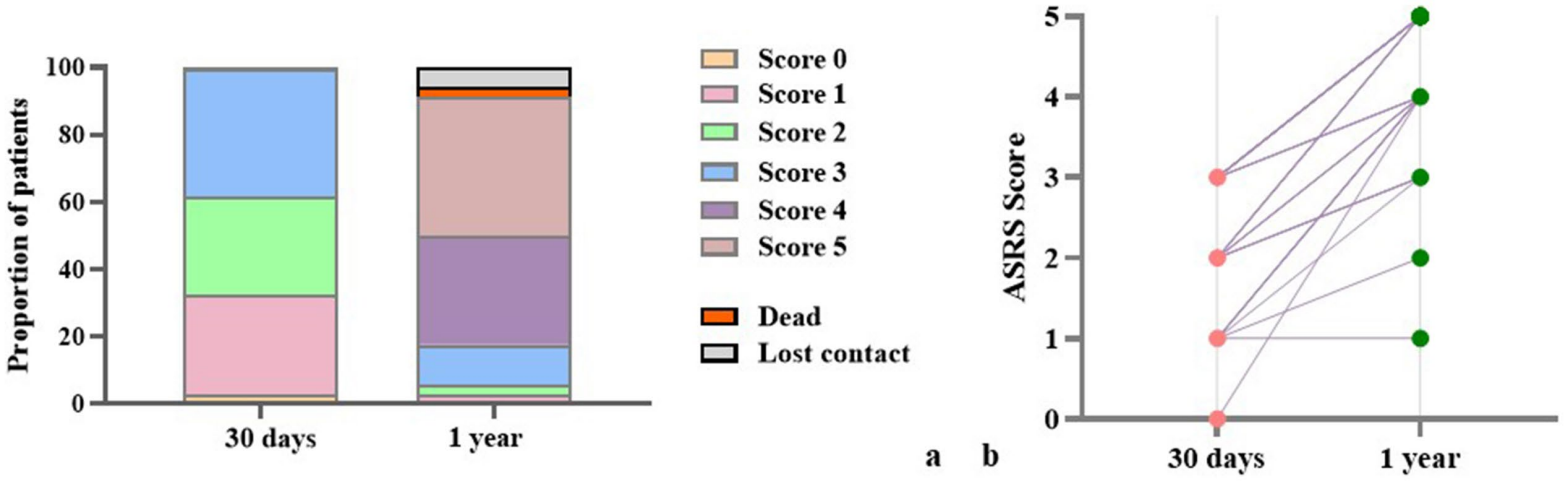
ASRS scores of 1 year follow-up patients. **(a)** Proportions of patients across ASRS score categories at 30 days and at 1 year post-stroke. **(b)** Individual-level changes in ASRS scores between 30 days and 1 year.

At the one-year follow-up, a small proportion of patients were lost to follow-up (5.9%), and two patients (2.9%) had died of stroke recurrence. Among those who completed the follow-up assessment, 73.6% of patients showed substantial language recovery, attaining ASRS scores of 4–5, indicative of near-normal language function (Table 1; Figure 5). These findings suggest a generally favorable long-term prognosis for subcortical aphasia, with the majority of patients regaining meaningful communicative function over time. Importantly, five patients had returned to their pre-stroke occupational roles (See Supplementary Table S1). To explore the potential linguistic characteristics of this subgroup, we compared their baseline language function with those of participants who did not return to work (non-RTW). At baseline, the return-to-work (RTW) group showed younger age and higher accuracy across PD, TD, SA tasks, the differences were statistically significant (See Supplementary Table S2). This finding indicates that baseline phonological and semantic performance are better in the RTW group than in the non-RTW group.

## Discussion

This study investigated the language characteristics of patients with subcortical aphasia using both standardized assessments and domain-specific language tasks. The findings revealed several key patterns that advance our understanding of the nature and structure of language impairment following subcortical stroke.

First, the distribution of aphasia subtypes in the cohort highlighted the predominance of fluent forms of aphasia, with anomic aphasia being the most frequent (47.1%), followed by Broca’s aphasia (29.4%). This finding aligns with prior studies suggesting that subcortical lesions, particularly those sparing key cortical speech areas, often result in less severe and more fluent aphasia presentations (6). Moreover, patients showed high WAB comprehension scores (*M* = 8.70), it could be seen that general aphasia assessment tool does not provide feedback on the deep-seated language impairments of subcortical aphasia patients.

Second, the results of the CALB-nb provided more granular insights into language processing deficits. The highest accuracy was observed in TD, while tasks requiring LfW and VAS were most challenging. LfW accuracy reflects patients’ ability to process low-frequency nouns, while VAS measures their performance across different argument structures. Both metrics are also associated with higher-order lexical-semantic processing abilities. These results suggest that while lower-level auditory perceptual abilities are often preserved, higher-order lexical-semantic processes may be disproportionately affected by subcortical damage. Previous studies have demonstrated that thalamic aphasia is predominantly characterized by disturbances in lexical-semantic processing, whereas language impairments resulting from basal ganglia lesions are more often associated with procedural dysfunctions, particularly those affecting the processing of syntactic and rule-governed language structures (26). In clinical populations, such as individuals with aphasia or dementia, language production and comprehension are often characterized by an increased reliance on high-frequency words and marked difficulty in accessing or recognizing low-frequency lexical items (6, 27). These findings suggest that patients with subcortical aphasia generally exhibit relatively preserved phonological input and output processes. However, their performance declines in tasks requiring higher-order language processing, such as visual/auditory object recognition and semantic association. This is most evident in their impaired ability to recognize and produce low-frequency nouns and their difficulty in using verbs with complex argument structures. This is consistent with the notion that subcortical structures contribute to lexical selection and semantic retrieval through their integrative and modulatory roles (12).

Correlation analyses further revealed significant positive relationships between multiple language domains. Notably, PO was strongly associated with both auditory perception and PD, emphasizing the interdependence between perceptual input and motor output pathways. Similarly, ALC showed strong correlations with both VAS and LfW, suggesting its central role in mediating access to semantic knowledge. This suggests that auditory comprehension is a complex procedure involving higher-order lexical-semantic operations. Furthermore, the extremely high correlations observed between NC and AE, as well as between LfW and VAS, point to semantic organization being a highly unified construct in these patients. This may reflect the distributed but interdependent nature of semantic representation, where impairments in one area are likely to be accompanied by deficits in another. In other words, semantic processing involves an interactive flow of information between processing stages, rather than a series of strictly discrete steps (28). Although *post hoc* power for medium effects was suboptimal (0.76), the actual effect sizes observed far exceeded this threshold, rendering the key findings statistically robust.

To further investigate the core linguistic deficits in subcortical aphasia, PCA was implemented to reduce dimensionality across linguistic variables. The results identified two key components accounting for 77.34% of the total variance. The first component, linked to *lexical and semantic processing*, explained 59.9% of the variance, while the second component, related to *phonological and auditory processing*, accounted for 17.4%. These findings indicate that subcortical aphasia may be conceptualized along two major cognitive-linguistic axes: one involving higher-order semantic and lexical access, and the other centered on lower-level phonological encoding and auditory discrimination. And the *lexical-semantic processing domain* consisted the primary source of linguistic heterogeneity among subcortical patients. The significant association between the composite PCA score and the AQ further confirms the clinical relevance of these dimensions, as they reflect overall aphasia severity. In other words, these results confirm that CALB assessment in subcortical aphasia patients can simultaneously capture both specific language domain impairments and global aphasia severity.

Importantly, our follow-up data indicated that 73.6% of patients achieved ASRS scores of 4–5 at 1 year, indicating near-normal communicative function. Five patients even returned to their pre-stroke employment. Further analysis of the RTW group revealed that these patients were generally younger and demonstrated better performance in phonological processing tasks. Although the current sample size was limited, these findings suggest the need for future studies with larger cohorts to investigate language-related prognostic factors.

Researchers have sought to elucidate the role of subcortical structures in aphasia through four proposed mechanisms: (1) direct involvement of subcortical nuclei in language processing (29); (2) disconnection between cortical language areas (30, 31); (3) subcortical lesion-induced hypoperfusion in perisylvian cortical regions (32); (4) diaschisis—remote neurophysiological changes resulting from a focal brain lesion (33, 34). However, language network analysis suggests a predominantly left-lateralized architecture, with subcortical structures such as the thalamus, putamen, and cerebellum frequently coactivated alongside cortical language areas, particularly the left pars opercularis (35). Broca’s area and other prefrontal regions are structurally connected to the ventral anterior nucleus of the thalamus (36). Through its language, the thalamus serves as a central integrative hub, linking the basal ganglia and cerebellum with premotor and prefrontal cortices via parallel circuits (37). Further evidence suggests that the thalamus selectively recruits cortical regions encoding multimodal lexical features and integrates them during lexical-semantic processing (12, 38). Diffusion-weighted tractography studies have demonstrated structural connectivity between prefrontal areas (including Broca’s area), the putamen (39) and the caudate nucleus (36). Functional imaging also indicates that the left putamen coactivates with frontotemporal regions during semantic tasks (40), while lesions in the left caudate are associated with impaired control of speech and language output (41). Collectively, these findings support the conceptualization of a cortico-striatal-thalamo-cortical loop, which facilitates lexical-semantic processing by enhancing activation of context-relevant semantic/phonological representations while suppressing competing alternatives (39). Although the present study did not employ lesion-symptom mapping or neuroimaging-based connectivity analyses, our behavioral results independently revealed a shared lexical-semantic impairment profile among patients with subcortical aphasia. Despite the inherent limitations of behavioral-only methods, the use of domain-specific, fine-grained neuropsychological assessments in this study provides valuable preliminary evidence and may offer a promising direction for future research integrating structural-functional connectivity frameworks.

The current study applied the CALB to Chinese-speaking populations, providing further evidence of profound language impairments in patients with subcortical aphasia, particularly in lexical and semantic processing. The CALB effectively captured aphasia severity, demonstrating its clinical utility as a routine assessment tool for subcortical aphasia. Its sensitivity in detecting subtle language disorders enables early identification of these impairments. Future research could integrate CALB evaluations with quantitative lesion-symptom mapping to further investigate these neural-language relationships.

In conclusion, our findings highlight the heterogeneous yet structured nature of language impairment in subcortical aphasia. Phonological, lexical, and semantic deficits often co-occur, but they can be decomposed into distinct dimensions that reflect overall language function. The results underscore the importance of multidimensional assessment tools and support a network-based model of language. These insights have direct implications for diagnosis, prognosis, and the design of targeted rehabilitation programs in aphasia care. Future study should focus on the tractography and cortical connectivity studies to explore the potential mechanism of subcortical lesions in language.

## Limitation

Nonetheless, the study has several limitations that warrant consideration. First, the sample size was relatively small. Although, the *post hoc* power analysis indicated adequate sensitivity for detecting medium effects, the limited sample size may still compromise the generalizability and robustness of the findings. Second, although the study employed rigorous inclusion criteria to focus exclusively on patients with subcortical aphasia following left-hemisphere stroke, it did not incorporate lesion-symptom mapping techniques to quantitatively assess the relationship between lesion location, lesion extent, and language performance. The lack of neuroimaging-based correlation restricts our ability to localize deficits to specific subcortical structures or networks. Integrating voxel-based lesion-symptom mapping or diffusion tensor imaging in future studies would provide critical anatomical specificity and further validate the behavioral findings. Third, the study relied solely on functional behavioral assessments without the support of high-resolution neuroimaging to elucidate the underlying neural mechanisms. While this approach ensures clinical feasibility, it may limit mechanistic interpretation and reduce alignment with contemporary neurocognitive models of language. Finally, the language outcomes at the 1-year follow-up were assessed using a relatively simplified tool, which may not have been sensitive enough to capture subtle or domain-specific changes in language function over time. Future longitudinal studies should consider incorporating more comprehensive, multidimensional assessment batteries to characterize the trajectory of language recovery in subcortical aphasia. Taken together, although this study contributes valuable insights into the lexical-semantic characteristics of subcortical aphasia using detailed behavioral profiling, future work should aim to expand the sample size, incorporate multimodal neuroimaging, and refine longitudinal assessment protocols to build a more comprehensive understanding of subcortical language impairments.

## Data Availability

The raw data supporting the conclusions of this article will be made available by the authors without undue reservation.
