# Supplementary material for: Characteristics and prognosis of language impairment in subcortical aphasia of acute stroke patients
Source: Front Neurol. 2025 Jul 16;16:1630365. doi: 10.3389/fneur.2025.1630365 (PMC12307362; doi:10.3389/fneur.2025.1630365)
Supplement: Supplementary file 1 [file Table_1.docx]

*Supplementary Material.*

**Full language evaluation procedure of CALB-nb**

Participants were assessed for CALB by speech therapist (S.L or X.C.) in the assessment room of the rehabilitation hall of Beijing Tiantan Hospital. All tasks are automatically initiated by tablet, and the therapists did not communicate with the patients (unless the patient had a sudden discomfort during the assessment, or had a specific request that needs to be answered).

If a participant was unable to press the buttons due to motor impairments, the therapists selected the corresponding response based on the participant’s reaction. To make sure that the tasks were well understood by all participants, they performed between 2 and 3 practice trials before starting the task. All tasks were presented auditorily and visually and thus required functional peripheral hearing and visual abilities, which are often impaired in older adults, such as those excluded in our study. The process for the8 subtests is as follows:

1) ***Auditory Discrimination.*** This task examines auditory processing ability for single words. Participants listened to two single-word pronunciations and determined whether the two words were auditorily identical. There were three practice trials and 22 test trials, among which seven pairs had identical pronunciations, six pairs differed in tone, four pairs differed in initial consonants, and four pairs differed in vowels. The practice trials were not included in the scoring. During the formal assessment, participants clicked on one of two areas on the screen: one with a checkmark representing "identical" and another with a cross representing "not identical." If participants did not make a selection within five seconds, their response was recorded as incorrect. If they indicated that they did not hear clearly or requested a repetition before making a judgment, the therapist was allowed to repeat the test item once.

Instruction："I will say two words in sequence. Please determine whether they sound the identical or not."

2) ***Tone recognition.*** This task examines participants' ability to recognize tones and retrieve corresponding object images from the semantic system. Participants listened to 12 pairs of high-frequency, commonly used, and highly imageable monosyllabic words. The target word and the distractor had identical consonant and vowel combinations but differed in tone, corresponding to different objects. The screen simultaneously displayed images corresponding to both the target and the distractor words, and participants selected the correct image after hearing the stimulus. Participants received each stimulus in turn, and had to categorize the sound as one of two options that were displayed on the screen.

3) ***Auditory Lexical Decision.*** This task assesses auditory processing ability for distinguishing between words and non-words. Participants listened to 16 two-syllable words and determined whether each was a real word or a pseudoword. Before the formal assessment, they completed two practice trials. If a participant failed to respond within five seconds or provided an incorrect response during practice, the trial was repeated once. If they failed again, the correct answer was provided.

Instruction: "I will say some words. Some are real Chinese words, such as 'péng -yǒu' (friend), and you should judge 'yes.' Some are non-existent words, such as 'lú-bào (not a real word),' and you should judge 'no.' I will say one word at a time, and you need to determine whether it is a real word."

4) ***Confrontation Naming.*** This task assesses the ability to name nouns and verbs. A total of 74 words from different categories were tested. The noun category effects were examined using items from two non-living categories (artifacts), i.e., tools and clothing, and two categories of living things, i.e., animals and fruits/vegetables. A fifth set of object nouns included only low-frequency items from various semantic categories. Additionally, naming of body parts and colors was tested. The action verbs tested included both transitive and intransitive forms. Participants were presented with an image and were required to name the object within 10 seconds. If they responded correctly, the next item followed. If they did not respond or provided an incorrect response, their answer was recorded.

Instruction: "What is this?"

5) ***Auditory Comprehension.*** This task assesses auditory lexical comprehension, consisting of 50 words categorized similarly to those in the Confrontation Naming task. Participants were presented with 10 images from the same category and were asked to select the corresponding image based on the given instruction.

Instruction: "Please point to the [target word]."

6) ***Semantic Association.*** This task assesses semantic processing ability. Participants were presented with 16 sets of object images primarily categorized as animals and tools. They were asked to select the pair of objects that were most semantically related.

Instruction: "Which pair of images is more related? You do not need to explain why, just make a selection."

7) ***Non-word Repetition.*** This task assesses the ability to repeat non-words. Participants listened to eight two-syllable non-words and repeated them after hearing the stimulus. If they indicated that they did not hear clearly or requested a repetition before responding, the therapist was allowed to repeat the test item once. However, if the participant had already started responding, no further repetition was permitted. Accurate repetition was required for a correct response; otherwise, it was marked as incorrect.

Instruction: “I will say some non-existent, meaningless words. I will say one word at a time, and after I finish, please repeat the word accurately."

8) ***Word Repetition.*** This task assesses the ability to repeat real words. Participants listened to 16 two-syllable words and repeated them after hearing the stimulus. The implementation method was identical to that of the Non-word Repetition task.

Instruction: "I will say some words, one at a time. After I finish, please repeat the word accurately."

Figure S1. Baseline language information for five patients returning to work.

| Characteristic | P01 | P02 | P03 | P04 | P05 |
| --- | --- | --- | --- | --- | --- |
| Occupation | construction worker | government officials | salesperson | primary school teacher | government officials |
| Age, year | 52 | 30 | 40 | 41 | 40 |
| AQ | 87.0 | 21.9 | 70.0 | 82.8 | 89.2 |
| Vision perception | 89 | 100 | 91 | 100 | 100 |
| Auditory perception | 96 | 0 | 100 | 100 | 100 |
| PD | 96 | 76 | 91 | 96 | 93 |
| TD | 88 | 100 | 100 | 100 | 100 |
| ALC | 81 | 77 | 84 | 97 | 97 |
| PO | 95 | 33 | 90 | 95 | 98 |
| PLP | 74 | 0 | 76 | 88 | 95 |
| LfW | 47 | 33 | 60 | 67 | 80 |
| NC | 85 | 29 | 87 | 97 | 99 |
| AE | 88 | 35 | 90 | 100 | 100 |
| NVR | 1.24 | 1.19 | 1.19 | 1.11 | 1.03 |
| VAS | 74 | 29 | 71 | 90 | 97 |
| SA | 81 | 69 | 63 | 100 | 100 |
| Pre-ASRS | 3 | 3 | 3 | 3 | 3 |
| Post-ASRS | 5 | 5 | 5 | 5 | 5 |

Abbreviation: Aphasia Quotient (AQ), Phonemic Decoding (PD), Tone Decoding (TD), Auditory Lexical Comprehension (ALC), Phonological Output (PO), Phonological Lexical Production (PLP), Low-frequency Word performance (LfW), Noun Categorization (NC), Animacy Effects (AE), Noun-to-Verb Ratio (NVR), Verb Argument Structure (VAS), and Semantic Association (SA).

Figure S2. Comparison of language function results between the RTW group and the non-RTW group.

| Characteristic | RTW group | Non-RTW group | *p*-value |
| --- | --- | --- | --- |
| Age, years, mean±SD | 40.60±7.80 | 54.45±9.61 | 0.006 |
| AQ, mean±SD | 70.58±28.35 | 56.12±21.05 | 0.138 |
| Vision perception,  median (IQR) | 96.0 (10.0) | 98.0 (25.5) | 0.406 |
| Auditory perception,  median (IQR) | 100.0 (52.0) | 92.0 (21.0) | 0.084 |
| PD, median (IQR) | 93.0 (12.5) | 80.0 (35.5) | 0.025 |
| TD, median (IQR) | 100.0 (6.0) | 85.0 (27.0) | 0.015 |
| ALC, median (IQR) | 84.0 (22.0) | 79.0 (20.0) | 0.131 |
| PO, median (IQR) | 95.0 (35.0) | 80.0 (36.0) | 0.118 |
| PLP, median (IQR) | 76.0 (54.5) | 72.0 (29.0) | 0.495 |
| LfW, median (IQR) | 60.0 (33.5) | 47.0 (53.5) | 0.626 |
| NC, median (IQR) | 87.0 (41.0) | 76.0 (36.5) | 0.330 |
| AE, median (IQR) | 90.0 (39.5) | 79.0 (36.0) | 0.284 |
| NVR, median (IQR) | 1.2 (0.1) | 1.3 (0.8) | 0.151 |
| VAS, median (IQR) | 74.0 (43.5) | 58.0 (44.5) | 0.144 |
| SA, median (IQR) | 81.0 (34.0) | 63,0 (34.0) | 0.023 |

Abbreviation: Return-to-work (RTW), Standard Deviation (SD), Aphasia Quotient (AQ), interquartile range (IQR), Phonemic Decoding (PD), Tone Decoding (TD), Auditory Lexical Comprehension (ALC), Phonological Output (PO), Phonological Lexical Production (PLP), Low-frequency Word performance (LfW), Noun Categorization (NC), Animacy Effects (AE), Noun-to-Verb Ratio (NVR), Verb Argument Structure (VAS), and Semantic Association (SA).
